# Sustainable and Low Viscous 1-Allyl-3-methylimidazolium Acetate + PEG Solvent for Cellulose Processing

**DOI:** 10.3390/polym9020054

**Published:** 2017-02-16

**Authors:** Airong Xu, Quan Li

**Affiliations:** School of Chemical Engineering & Pharmaceutics, Henan University of Science and Technology, Luoyang 471003, Henan, China; liquanhenan@126.com

**Keywords:** [Amim][CH_3_COO]/PEG solvent, sustainability, cellulose, dissolution mechanism, regenerated cellulose properties

## Abstract

Developing sustainable, low viscous and efficient solvents are always advantageous to the processing/fabricating of cellulose materials in practical applications. To this end, in this work novel solvents were developed; ([Amim][CH_3_COO]/PEG) by dissolving polyethylene glycol 200 (PEG-200) in 1-allyl-3-methylimidazolium acetate ([Amim][CH_3_COO]). The solubilities of cellulose in [Amim][CH_3_COO]/PEG solvents were determined as a function of temperature, and the possible dissolution mechanism of cellulose in [Amim][CH_3_COO]/PEG solvent was investigated. The novel solvent exhibits outstanding advantages for good dissolution capacity of cellulose, such as low viscosity, negligible vapor pressure, and recycling capability. The [CH_3_COO]^−^ anion and the [Amim]^+^ cation of [Amim][CH_3_COO] in [Amim][CH_3_COO]/PEG-10 are the driving force for cellulose dissolution verified by the ^13^C NMR spectra. In addition, the regenerated cellulose films from [Amim][CH_3_COO]/PEG solvent were characterized by scanning electron microscopy (SEM), X-ray diffraction (XRD), attenuated total reflectance Fourier transform infrared spectroscopy (ATR-FTIR), and thermogravimetric analysis (TGA) to estimate their morphologies and structures.

## 1. Introduction

With the increasing depletion of petroleum resources, the exploitation of low-cost biorenewables, bioresource has received great attention [1,2]. Cellulose, as the most abundant renewable resource in nature, has been regarded as a potential alternative to fossil resource. However, cellulose is extremely difficult to dissolve in water and most conventional organic solvents due to the close packing by numerous inter- and intra-molecular hydrogen bonds. Therefore, many possible applications of this cheap and abundant natural resource are limited. To tackle the issue, great efforts have been made in the scientific community in recent years, and a series of cellulose solvents have been developed such as *N*-methylmorpholine-*N*-oxide, lithium chloride + *N*,*N*-dimethylacetamide, tetrabutyl ammonium fluoride + dimethyl sulfoxide DMSO, NaOH/Thiourea, etc. [3,4,5,6].

In recent years, ionic liquids (ILs), especially imidazolium-based carboxylate ILs have received considerable attention due to their powerful capacity for cellulose dissolution [7,8,9,10,11,12]. Recently, some efforts have been made to develop more efficient cellulose solvent systems by adding co-solvents to ILs. For example, Rinaldi developed solvent systems (1-butyl-3-methylimidazolium chloride + aprotic polar solvent including dimethyl sulfoxide DMSO, *N*,*N*-dimethylformamide DMF, etc.) which have lower viscosity and higher dissolving rate than neat ILs [13]. Later on, Xu et al. found that 1-butyl-3-methylimidazolium acetate [Bmim][CH_3_COO] + DMSO (DMF or *N*,*N*-dimethylacetamide DMAc) solvents could effectively dissolve cellulose at ambient temperature without any heating [14,15,16]. Sun et al. developed tetrabutylammonium acetate/dimethyl sulfoxide (TBAA/DMSO) solvent which could dissolve cellulose and spin cellulose fibers by a wet spinning system [17]. Rein et al. investigated the structure of cellulose in 1-ethyl-3-methylimidazolium acetate [Emim][CH_3_COO] + DMSO/DMF solvent and found that cellulose was dissolved molecularly in the solvents [18]. 

It has been reported that 1-allyl-3-methylimidazolium acetate [Amim][CH_3_COO] displays powerful dissolution capacity for cellulose even at ambient temperature [8]. Nevertheless, neat [Amim][CH_3_COO] is slightly viscous which is not conducive to cellulose dispersion despite less viscosity than the chloride/bromide-based IL counterparts [19]. As mentioned above, the addition of aprotic polar solvent to IL can significantly not only improve cellulose solubility but reduce solvent viscosity and thus improve the mass transport and kinetics of dissolution. However, aprotic polar solvents are volatile organic compounds which are easily lost to the environment and cause environmental pollution. Moreover, the aprotic polar solvents + IL solvents are difficult to recover and recycle. To tackle the issue, adding a cosolvent with negligible vapor pressure instead of a volatile organic solvent is a promising strategy.

Therefore, in the present work, novel [Amim][CH_3_COO]/PEG solvents were developed by adding PEG-200 to [Amim][CH_3_COO]. The selection of PEG-200 is based on the fact that, PEG-200 is a nonvolatile, biodegradable, corrosion inhibiting, cheap and easily obtained molecular solvent, which has been approved by the FDA for internal consumption. Moreover, PEG was reported to be a hydrogen-bonding acceptor that prevents the re-association of hydroxyl groups of cellulose forming gel, and the chains of the PEG make its repeat units difficult to be extruded out from solution compared with small molecules such as urea or thiourea, by the self-association of cellulose molecules, which results in the stability of the cellulose solution [20]. At the same time, the solubilities of cellulose in [Amim][CH_3_COO]/PEG solvents were determined. ^13^C NMR technique was employed to investigate the possible dissolution mechanism of cellulose in [Amim][CH_3_COO]/PEG solvent. Additionally, the regenerated cellulose films from [Amim][CH_3_COO]/PEG solvent were characterized by scanning electron microscopy (SEM), X-ray diffraction (XRD), attenuated total reflectance Fourier transform infrared spectroscopy (ATR-FTIR), and thermogravimetric analysis (TGA). 

## 2. Experimental Section

### 2.1. Materials 

Microcrystalline cellulose (MCC) with a 270 viscosity-average degree of polymerization (DP) was purchased from Sigma Aldrich Company (Shanghai, China). Polyethylene glycol (PEG) with a number-average molecular weight of 200 was purchased from Shanghai Jingchun Biotechnology Co. Ltd. (Shanghai, China). Deuterated DMSO (DMSO-*d*_6_) (>99.9%) used for NMR samples was purchased from Qingdao Weibo Tenglong Technology Co., Ltd. (Qingdao, China). The above materials were used as received without further purification. [Amim][CH_3_COO] was synthesized and purified by using the procedure described in the literature [14].

### 2.2. Dissolution of Cellulose in [Amim][CH_3_COO]/PEG Solvents

[Amim][CH_3_COO]/PEG-10 and [Amim][CH_3_COO]/PEG-20 containing 10 wt % and 20 wt % PEG in [Amim][CH_3_COO]/PEG solvent, respectively were prepared by adding PEG in dried [Amim][CH_3_COO] under stirring. Cellulose was added into a 25 mL colorimetric tube which contained 2.0 g of [Amim][CH_3_COO]/PEG, and the tube was sealed with parafilm. The tube was then immersed in an oil bath (DF-101S, Gongyi Yingyu Instrument Factory, Gongyi, China), and the instability of the bath temperature was estimated to be ±0.5 °C. After the cellulose was fully dissolved, the solution became completely clear and no cellulose particles were observed under a polarization microscope (Nanjing Jiangnan Novel Optics Co. Ltd., Nanjing, China). Then, additional cellulose was added. When the cellulose solution became saturated to the point that no more cellulose could be dissolved, its solubility (expressed by gram per 100 g of solvent) at a given temperature was calculated from the amount of the solvent and cellulose added. The solubilities of cellulose in [Amim][CH_3_COO]/PEG solvents are summarized Table 1. 

### 2.3. Measurements ^13^C NMR Spectra

Measurements of ^13^C NMR spectra for [Amim][CH_3_COO] in [Amim][CH_3_COO]/PEG-10 solvent, [Amim][CH_3_COO]/PEG-20 solvent, [Amim][CH_3_COO]/PEG-10/cellulose solution were performed on a Bruker DMX 300 spectrometer (Bruker Corporation, Rheinstetten, German) at room temperature. DMSO**-***d*_6_ was used as an external standard. Chemical shifts were given in ppm downfield from TMS. An [Amim][CH_3_COO]/PEG-10/cellulose solution with 8.0% solubility was obtained by dissolving cellulose in [Amim][CH_3_COO]/PEG-10 solvent. 

### 2.4. Preparation and Characterization of Regenerated Cellulose Film

Five percent of cellulose solution was prepared by dissolving cellulose in [Amim][CH_3_COO]/PEG-10 solvent at 40 °C. The solution was cast onto a glass plate to give a thickness of about 2 mm, the air bubbles removed in a vacuum oven for 30 min, and then immediately coagulated in water to obtain a transparent regenerated cellulose gel film. The regenerated cellulose gel film was washed with running distilled water followed by drying at 60 °C in a vacuum oven. The dried cellulose film was coded as RC-A.

The above as-prepared cellulose gel film was frozen for 2 h in a refrigerator, then freeze-dried using a FD-10 freeze-dryer (Henan Xiongdi Instrument Equipment Co. Ltd., Zhengzhou, China). The cold trap temperature was below −45 °C and the vacuum pressure was below 0.1 MPa during the freeze-drying process. The freeze-dried cellulose film was coded as RC-F. 

Five percent of as-prepared cellulose solution was cast onto a glass plate to give a thickness of about 2 mm, air bubbles removed in a vacuum oven for 30 min. Then, the cellulose solution film was frozen for 2 h at –80 °C and subsequently immersed in distilled water for regeneration-gelation. The gel was repeatedly washed by distilled water to remove [Amim][CH_3_COO]/PEG. After being frozen for 2 h in a refrigerator, the hydrogel was freeze-dried using a FD-10 freeze-dryer to obtain cellulose aerogel. The cold trap temperature was below −45 °C and the vacuum pressure was below 0.1 MPa during the freeze-drying process. The freeze-dried cellulose film was coded as RC-FF. 

The RC-A, RC-F, and RC-FF films were employed for the measurements of SEM, XRD, ATR-FTIR spectroscop, and TGA. Scanning electron micrographs (SEM) of the regenerated cellulose films in the dry state were frozen in liquid nitrogen, and immediately snapped. The fractured surfaces of the films were sputtered with gold, and then photographed. The XRD patterns were collected on a BrukerD8Advance diffraction spectrometer with Cu-Ka radiation (*λ* = 1.54 Ǻ) over the range 3°–60° (2θ) at a scan speed of 2° (2θ) per minute. An ATR-FTIR (Nicolet iN10, Thermo Fisher Scientific, USA) system with Ge crystal ATR accessory, MCT (mercury–cadmium telluride) detector, and OMINC picta workstation (Thermo Fisher Scientific, Waltham, MA, USA) were employed for IR observation. Spectra were collected in high-resolution mode (4 cm^–1^ resolution and 64 scans) under ATR 5% maximum pressure. Background was subtracted for every measurement. Triplicate tests were performed at different sites for every sample. Thermogravimetric analysis (TGA) was carried out with a NETZSCH STA 449 C thermal analyzer (Netzsch Corporation, Freistaat Bayern, German) using alumina crucibles. The measurements were carried out under flowing N_2_ at a heating rate of 10 °C·min^−1^. 

## 3. Results and Discussion

### 3.1. Dissolution Behavior of Cellulose in [Amim][CH_3_COO]/PEG Solvent

It is well-known that low viscous solvents improve mass transport and enable faster kinetics of dissolution, which facilitates cellulose processing. It has been reported that the viscosity of PEG (48.2–58.8 mPa·s) is considerably less than that of [Amim][CH_3_COO] (88.9 mPa·s) [19,21,22], Therefore, after the addition of PEG to [Amim][CH_3_COO], [Amim][CH_3_COO]/PEG solvent is less viscous compared to [Amim][CH_3_COO], which was experimentally observed. 

Dissolution behavior of cellulose in the [Amim][CH_3_COO]/PEG solvents was examined, and the solubility values of cellulose are shown in Table 1. It can be seen that the PEG content in [Amim][CH_3_COO]/PEG solvent remarkably impacts cellulose solubility. The [Amim][CH_3_COO]/PEG-10 solvent displays the strongest dissolution capacity of cellulose compared with [Amim][CH_3_COO]/PEG-20 and [Amim][CH_3_COO]/PEG-30 solvents. For example, high cellulose solubility (10.2% in [Amim][CH_3_COO]/PEG-10) can be acquired at 40 °C. However, a dramatic decrease in cellulose solubility is observed when the PEG content increases to 20 wt %, when only 2.0% cellulose solubility is obtained in [Amim][CH_3_COO]/PEG-20 solvent at 40 °C. Furthermore, when the PEG content in [Amim][CH_3_COO]/PEG solvent increases to 30 wt %, cellulose does not dissolve. 

### 3.2. Possible Dissolution Mechanism of Cellulose in [Amim][CH_3_COO]/PEG Solvent

**Figure 1:**
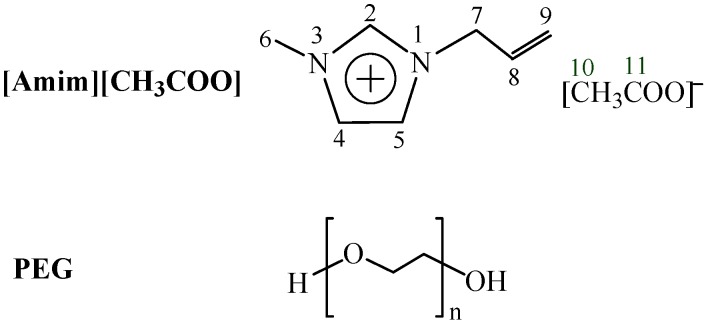
Schematic structure and carbon numbering of [Amim][CH_3_COO] (**upper**) and PEG (**below**).

[Amim][CH_3_COO] has been reported to be a powerful solvent for cellulose, and [Amim^+^ and [CH_3_COO]^−^ of [Amim][CH_3_COO] dominate the dissolution of cellulose [8]. PEG is unable to dissolve cellulose. Therefore, [Amim^+^ and [CH_3_COO]^–^ of [Amim][CH_3_COO] is the determining factor for the dissolution behavior of the [Amim][CH_3_COO]/PEG solvent. To verify the assumption, the ^13^C NMR spectra of [Amim][CH_3_COO] in [Amim][CH_3_COO]/PEG-10 solvent and [Amim][CH_3_COO]/PEG-10/cellulose (8%) solution were determined at room temperature and shown in Figures S1 and S2 (see Supplementary Materials). The ^13^C NMR data of [Amim][CH_3_COO] are given in Table 2. For the sake of easy understanding, the schematic structures and numberings of C atoms of [Amim][CH_3_COO] and PEG are shown in Figure 1.

As cab be seen in Table 2, after the addition of cellulose to [Amim][CH_3_COO]/PEG-10 solvent, the signal of the C11 atom in [CH_3_COO]^–^ anion is significantly downfield (an increase of chemical shift), suggesting that the oxygen atom in [CH_3_COO]^–^ anion forms a hydrogen bond with the hydroxyl hydrogen atom of cellulose, which results in the decrease of the electron cloud density of the C11, thus its chemical shift moves downfield. It is also observed that, the signals of the C2, C4, C5, and C8 atoms in [Amim]^+^ cation considerably move upfield, implying that these hydrogen atoms in the [Amim]^+^ cation interact with the oxygen atoms of cellulose through hydrogen bond formation, which causes an increase of the electron cloud density in these carbon atoms, thus their chemical shift moves upfield. Additionally, the marked upfield shifts of C9 and C10 may result from the redistribution of the electron cloud density around these carbon atoms after the hydrogen bond interactions. Few changes are observed for the chemical shifts of C6 and C7 in [Amim]^+^ cation. The findings are consistent with the assumption aforementioned that the [CH_3_COO]^–^ anion and [Amim]^+^ cation of [Amim][CH_3_COO] in [Amim][CH_3_COO]/PEG-10 are the driving force for cellulose dissolution.

### 3.3. Recovery of [Amim][CH_3_COO]/PEG Solvent

Recovery of solvent not only means sustainable production but also reduces costs and possible harm to the environment in practical application. As such, the renewability of [Amim][CH_3_COO]/PEG solvent was estimated. To recover [Amim][CH_3_COO]/PEG-10 solvent, 6.0 g of [Amim][CH_3_COO]/PEG-10 solvent and 5.0 wt % cellulose solution were used. The cellulose solution was poured into a 100 mL beaker containing 20 mL of water. The mixture in the beaker was stirred for 30 min at ambient temperature. The precipitated cellulose was separated by filtration through a ceramic funnel under vacuum. The cellulose was washed four times to ensure that [Amim][CH_3_COO]/PEG-10 had been washed out. The filtrates were combined in a round bottomed flask, and water was removed by rotatory evaporation under reduced pressure. The resultant [Amim][CH_3_COO]/PEG-10 solution was dried under vacuum for 24 h at 55 °C, and then used in the next dissolution process. In each dissolution–recovery cycle, the recovery percentage of [Amim][CH_3_COO]/PEG-10 solvent is approximately 98 wt %, and the dissolving capacity of the recovered solvent for cellulose is equivalent to the original solvent. Moreover, the ^13^C NMR spectra of the recovered solvent is in agreement with that of the original solvent (see Figures S3 and S4), indicating that there are no chemical reactions between cellulose and [Amim][CH_3_COO]/PEG, and the dissolution of cellulose in [Amim][CH_3_COO]/PEG is a pure physical process.

### 3.4. Properties of the Regenerated Cellulose Films

SEM, XRD, ART-IR spectroscopy, and TGA were used to characterize the original cellulose and the cellulose films regenerated from [Amim][CH_3_COO]/PEG-10/cellulose solution. 

**Figure 2:**
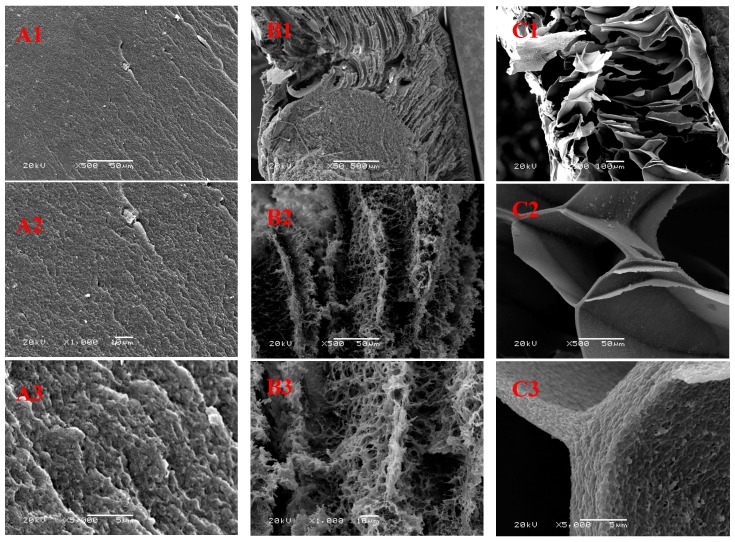
Scanning electron microscopy (SEM) images of the fracture surfaces of cellulose films; RC-A at 500× magnification (**A1**), 1000× magnification (**A2**) and 5000× magnification (**A3**), respectively, RC-F at 50× magnification (**B1**), 500× magnification (**B2**) and 1000× magnification (**B3**), respectively, RC-FF at 100× magnification (**C1**), 500× magnification (**C2**) and 5000× magnification (**C3**), respectively.

Figure 2 shows SEM images of the fracture surfaces of RC-A, RC-F, and RC-FF cellulose films. Compared with the RC-F and RC-FF cellulose films, the RC-A cellulose film displays a homogeneous dense structure. The RC-F cellulose film exhibits a unidirectional long porous structure. Moreover, the sheets of the long porous structures are fluffy and porous. The RC-FF cellulose film shows fluffy and porous architectures in which the porous structure is composed of randomly oriented cellulose sheets, and the sheets are twisted and broken. A comparison of the RC-A, RC-F, and RC-FF cellulose films reveals that the architecture of the regenerated cellulose material can be modified by changing the cellulose processing strategies.

**Figure 3:**
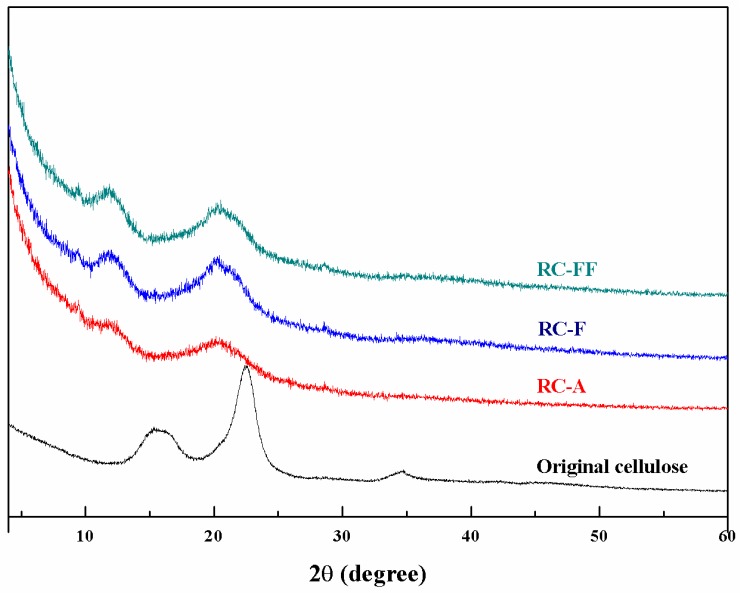
X-ray diffraction (XRD) spectra of RC-A, RC-F, and RC-FF, and the original cellulose.

The XRD patterns of the original cellulose, RC-A, RC-F, and RC-FF cellulose films are shown in Figure 3. The original cellulose is cellulose I as indicated by the typical diffraction peaks at 2θ = 15.2°, 16.4°, 22.5°, 34.6° [23]. The regenerated RC-A, RC-F, and RC-FF cellulose films exhibit the same diffraction peaks and the typical diffraction patterns of cellulose II at 2θ = 12.5°, 20.3° and 21.2° [24]. This indicates that the transformation from cellulose I to cellulose II occurred after a series of processing steps such as dissolution, regeneration, freezing, or/and freeze-drying. 

**Figure 4:**
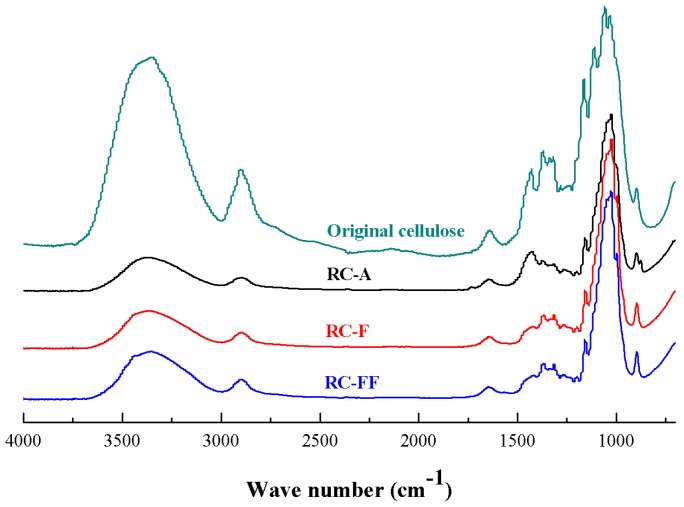
Fourier transform infrared spectroscopy (FT-IR) spectra of of RC-A, RC-F, and RC-FF, and the original cellulose.

ATR-FTIR spectra of the original cellulose and the regenerated RC-A, RC-F, and RC-FF cellulose films are shown in Figure 4. Clearly, the spectra of the original cellulose and the regenerated cellulose films are quite similar, and no new peaks are observed in the regenerated cellulose film sample. This implies that no chemical reaction occurs between cellulose and [Amim][CH_3_COO]/PEG solvent during the dissolution and regeneration processes of the cellulose, which is in accordance with the result aforementioned. The absorption band at 1423 cm^–1^ in the regenerated cellulose film is assigned to the CH_2_ scissoring vibration. This band was weakened and shifted to a lower wavenumber compared to the peak at 1431 cm^–1^ for the original cellulose, suggesting the destruction of an intra-molecular hydrogen bond involving O6 [25]. A new shoulder at 990 cm^–1^ in the regenerated cellulose film could be assigned to the C–O stretching vibration in the amorphous region [26] The O–H vibration in the regenerated cellulose film shifts to a higher wavenumber (3370 cm^–1^), which is an indication of the breaking of hydrogen bonds to some extent [27,28]. The absorption bands in the range of 1164–1061 cm^–1^ belong to the C–O–C stretching of the original cellulose [29]. The presence of such bands in the absorption of the regenerated cellulose suggests that the macromolecular structure of cellulose is not destroyed after regeneration of the cellulose. 

**Figure 5:**
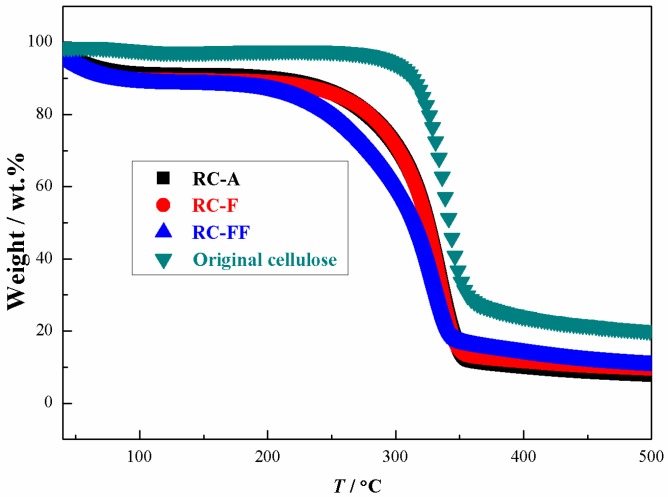
Thermal decomposition profiles of RC-A, RC-F, and RC-FF, and the original cellulose.

TGA curves for the original cellulose, RC-A, RC-F, and RC-FF cellulose films are shown in Figure 5. The regenerated cellulose films exhibit a slightly lower onset temperature (298 °C for RC-A and RC-F; 283 °C for RC-FF) for the decomposition compared to the original cellulose (323 °C), and give a slightly lower char yield (nonvolatile carbonaceous material) on pyrolysis, indicated by the slightly lower residual mass after the decomposition step. This indicates that the cellulose regenerated from the [Amim][CH_3_COO]PEG-10 solvent has good thermal stability. 

## 4. Conclusions

Low viscous and efficient solvents ([Amim][CH_3_COO]/PEG) have been developed, which are advantageous for processing/fabricating cellulose materials in practical applications. Moreover, the solvents have negligible vapor pressures and can be recycled, which can enable sustainable production, lower energy consumption and costs, as well as reducing possible harm to the environment. The possible dissolution mechanism for cellulose is suggested to be that the [CH_3_COO]^–^ anion and [Amim]^+^ cation of [Amim][CH_3_COO] in [Amim][CH_3_COO]/PEG solvent mainly contribute to cellulose dissolution. By changing cellulose processing strategies, the architecture structure of the regenerated cellulose material can be modified. Moreover, there are no chemical reactions between lignin and [Amim][CH_3_COO]/PEG. In addition, TGA findings indicate that the regenerated cellulose exhibits good thermal stability compared to the original cellulose.

## Figures and Tables

**Table 1:** Solubility of microcrystalline cellulose in the ILs at different temperatures.

Solvent	Solubility (gram per 100 g of solvent)				
	30 °C	40 °C	50 °C	60 °C	70 °C
[Amim][CH_3_COO]	14	16.0	16.5	18.0	21.0
[Amim][CH_3_COO]/PEG-10	2.9	10.2	11.2	11.4	11.8
[Amim][CH_3_COO]/PEG-20	0.9	2.0	5.0	5.5	5.8
[Amim][CH_3_COO]/PEG-30	Insoluble	Insoluble	Insoluble	Insoluble	Insoluble

**Table 2:** The ^13^C NMR chemical shifts (*δ* (ppm) relative to TMS) of [Amim][CH_3_COO] in [Amim][CH_3_COO]/PEG-10 solvent and [Amim][CH_3_COO]/PEG-10/cellulose(8%) solution at room temperature.

Cellulose concentration (%)	C2	C4	C5	C6	C7	C8	C9	C10	C11
0	137.94	122.54	123.91	35.34	50.45	132.24	119.43	25.17	175.16
8	137.28	122.31	123.79	35.46	50.56	131.82	119.79	24.70	175.94
∆*δ*	–0.66	–0.23	–0.12	0.12	0.11	–0.42	0.36	–0.47	0.78

## Supplementary Materials

The following are available online at www.mdpi.com/2073-4360/9/2/54/s1. Figure S1: ^13^C NMR spectra of [Amim][CH_3_COO] in [Amim][CH_3_COO]/PEG-10 solvent at room temperature, Figure S2: ^13^C NMR spectra of [Amim][CH_3_COO] in [Amim][CH_3_COO]/PEG-10/cellulose(8%) solution at room temperature, Figure S3: ^13^C NMR spectra of the original [Amim][CH_3_COO]/PEG-10 solvent at room temperature, Figure S4: ^13^C NMR spectra of the recovered [Amim][CH_3_COO]/PEG-10 solvent at room temperature.
