# Supplementary material for: Sustainable and Low Viscous 1-Allyl-3-methylimidazolium Acetate + PEG Solvent for Cellulose Processing
Source: Polymers (Basel). 2017 Feb 16;9(2):54. doi: 10.3390/polym9020054 (PMC6432357; doi:10.3390/polym9020054)
Supplement: Supplementary file 1 [file polymers-09-00054-s001.pdf]

Supplementary Materials: Sustainable and Low Viscous 1-Allyl-3-methylimidazolium Acetate + PEG Solvent for Cellulose Processing

Airong Xu and Quan Li

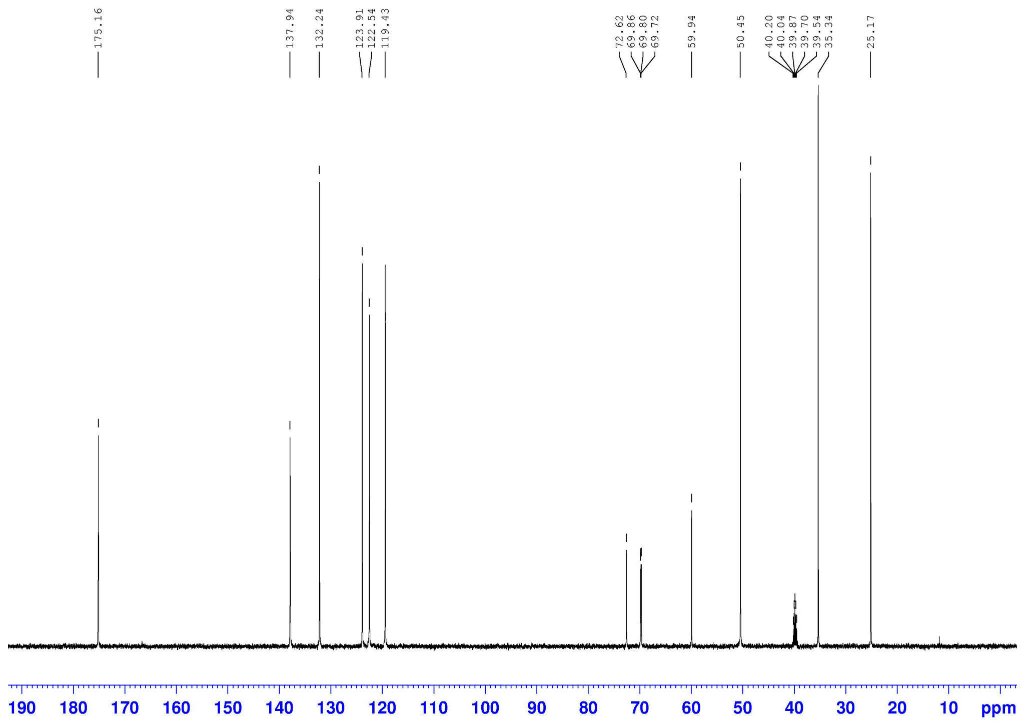

Figure S1. ^{13}C NMR spectra of [Amim][CH_3COO] in [Amim][CH_3COO]/PEG-10 solvent at room temperature.

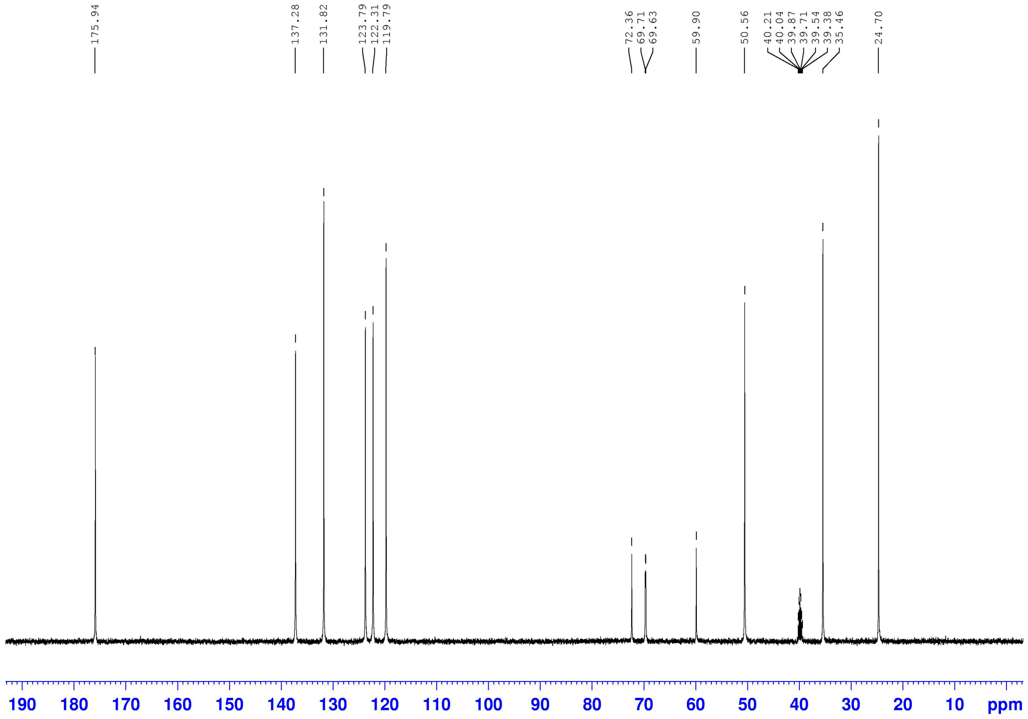

Figure S2. ^{13}C NMR spectra of [Amim][CH_3COO] in [Amim][CH_3COO]/PEG-10/cellulose(8%) solution at room temperature.

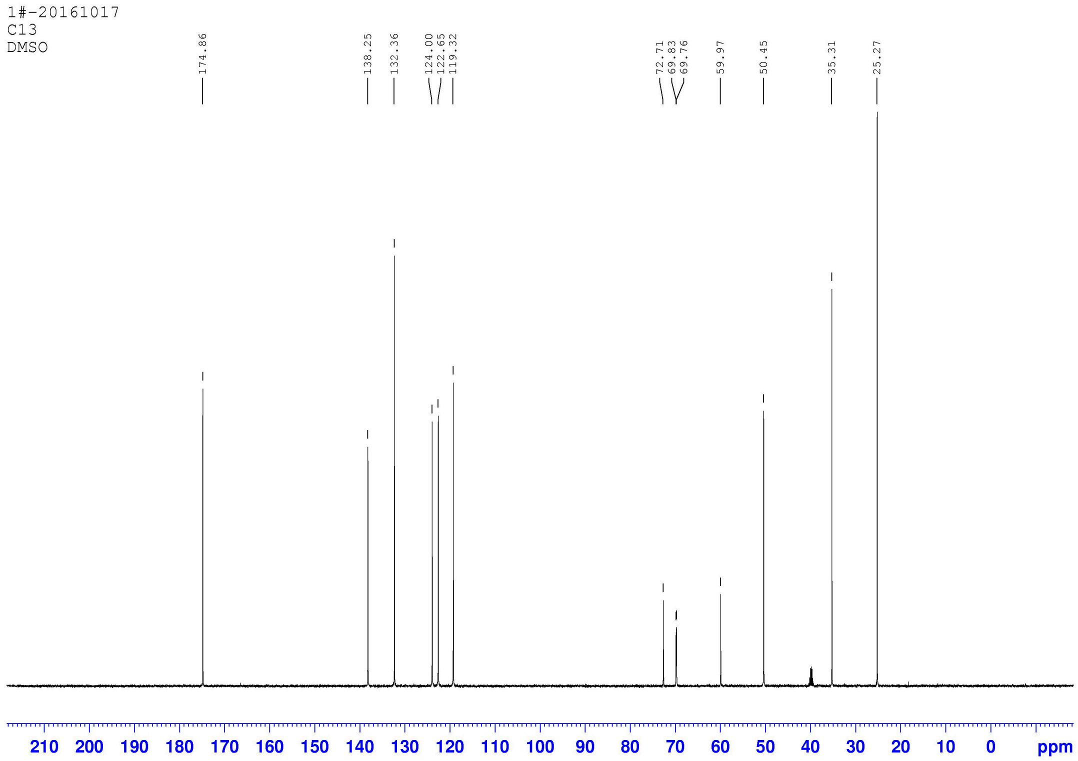

Figure S3. ^{13}C NMR spectra of the original [Amim][CH_3COO]/PEG-10 solvent at room temperature.

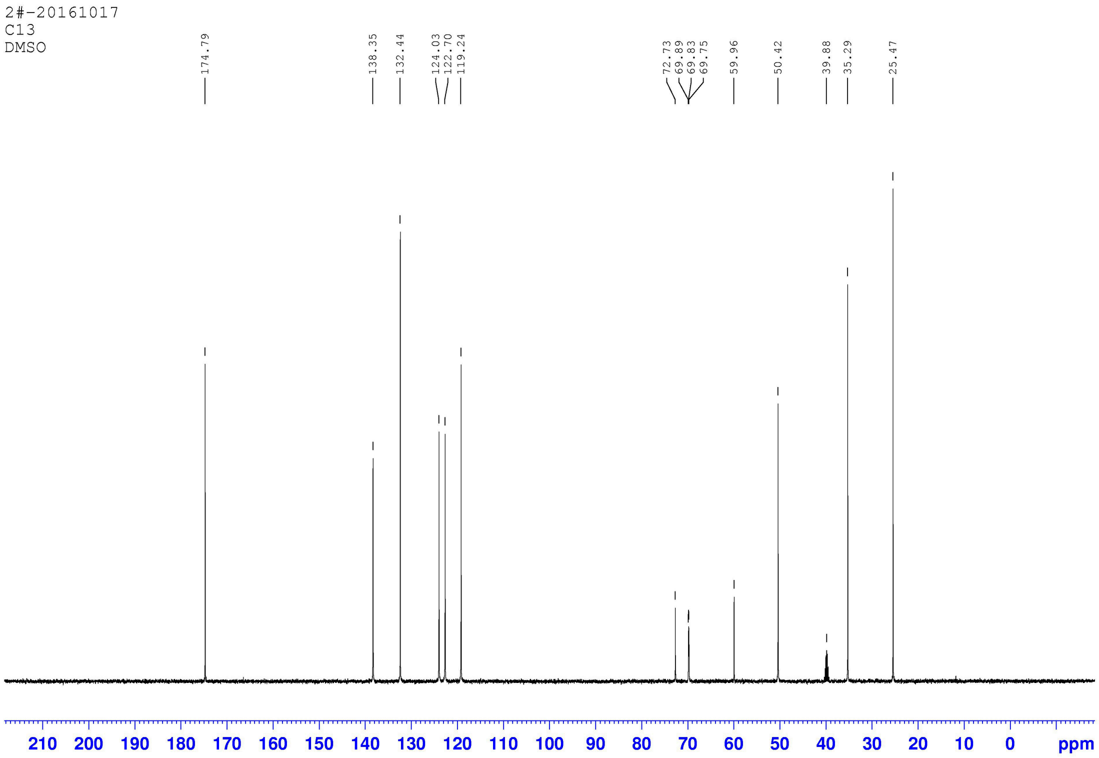

Figure S4. ^{13}C NMR spectra of the recovered [Amim][CH_3COO]/PEG-10 solvent at room temperature.
